# **GABA**_**A**_ **Receptors in the Dorsal Hippocampus are Involved in** **Sate-dependent Learning Induced by Lithium in Mice**

**Published:** 2011

**Authors:** Leila Parsaei, Mahya Rangchiyan, Shamseddin Ahmadi, Mohammad Reza Zarrindast

**Affiliations:** a *Department of Pharmacology and Iranian National Center for Addiction Studies, School of Medicine, Tehran University of Medical Sciences, Tehran, Iran*.; b *Department of Biological Science and Biotechnology, Faculty of Science, University of Kurdistan, Sanandaj, Iran.*; c *School of Cognitive Sciences, Institute for Studies in Fundamental Sciences (IPM), P.O. Box 19395-57463, Tehran, Iran.*; d *Institute for Cognitive Science Studies, Tehran, Iran.*

**Keywords:** Hippocampus, Bicuculline, Muscimol, State-dependent, Memory, Mice

## Abstract

These experiments examined the effects of pre-test intra dorsal hippocampus (intra-CA1) administration of GABA_A_ receptor agonist and antagonist, muscimol and bicuculline respectively, on state-dependent learning induced by lithium. Male NMRI mice were trained in a one-trial step-down inhibitory avoidance task, and immediately after training they received IP injections of either saline (10 mL/kg) or lithium (10 mg/kg). The animals were tested for step-down latency, as an index of inhibitory avoidance memory, 24 h after the training. The results showed that lithium (10 mg/kg) induced state-dependent learning. Although the decrease of step down latency due to post-training lithium (10 mg/kg) was not fully reversed by a lower dose of lithium (5 mg/kg) but pre-test intra-CA1 injection of bicuculline (0.25 μg/mouse) increased the effect of the lower dose of lithium. Pre-test intra-CA1 injection of muscimol (0.05 μg/mouse) by itself reversed the decrease of step-down latency induced by post-training lithium (10 mg/kg). Pre-test intra-CA1 injection of muscimol (0.03 and 0.05 μg/mouse) also disrupted state-dependent learning induced by lithium (10 mg/kg). The results suggest that a GABA_A_ receptor mechanism in the dorsal hippocampus is involved in state-dependent induced by lithium.

## Introduction

In the step down inhibitory avoidance task, animals learn to avoid stepping down from a platform on a grid floor where they once received a footshock. It has been reported that this task relies heavily on the dorsal hippocampus (1, 2). A number of studies have indicated that several neurotransmitter systems are involved in hippocampl-dependent learning and memory (1, 3).

Gama-aminobutyric acid (GABA) is the main central inhibitory neurotransmitter which acts through the interaction with GABA_A_, GABA_B_ and GABA_C_ receptors (4-6). The GABA_A_ and GABA_C_ receptors are ionotropic which directly associated with a Cl^-^ channel (7), and mediate rapid synaptic inhibition (8, 9). The GABA_B_ receptors are coupled to the K^+^ channels via G proteins and mediate the late component of inhibitory postsynaptic potentials (6, 10, 11). GABA plays a controlling role on the balance of excitability and inhibitory states in the cortex and hippocampus (12). It has been reported that GABA_A_ receptors are responsible for mediating a wide range of activities, including anticonvulsant (13), sedation and hypnosis (14). It is also well known that the GABA_A_ receptors affect learning and memory processes (15, 16). GABA_A_ receptor agonists impair memory while their antagonists facilitate retrieval in different tasks (3, 15, 17-19).

Lithium has been used as an important mood stabilizing drug in the treatment of manic-depressive illness and can potentiate the effects of antidepressant drugs (20-22). In addition to its established role as a mood stabilizer, a neuroprotective (23, 24) and an antiapoptotic effect (25, 26) for lithium have been reported. Furthermore, clinical observations have suggested that lithium may exert adverse effects on memory. It has been reported that the drug treatment inhibited learning, memory, and speed of information processing in bipolar disorder patients and to some extent in control subjects (27-30). We have already reported a state-dependent learning induced by lithium (31-33). We have also reported a possible involvement of nitric oxide (31), *μ*-opioid receptors (34) and dopaminergic system (32) in lithium-induced state-dependent learning. Considering role of the dorsal hippocampus in mediating inhibitory avoidance memory (1), and involvement of a GABA signaling in the effect of lithium (35), this study was conducted to understand the involvement of GABA_A_ receptors of the dorsal hippocampus in state-dependent learning induced by lithium.

## Experimental

*Materials*

*Drugs*

The drugs used in the present study were lithium chloride (Merck, Germany), muscimol (Tocris, UK), bicuculline (Sigma, St. Louis, CA, USA). All drugs were dissolved in sterile 0.9% saline, just before the experiment, except for bicuculline that was dissolved in one drop of glacial acetic acid and made up to a volume of 2 mL with sterile 0.9% saline and then diluted to the required volume. Muscimol and bicuculline were injected into the CA1 regions of the dorsal hippocampi (intra-CA1) and lithium was injected intraperitoneally (IP). Control animals received either saline and/or vehicle.

*Methods*

*Subjects*

Male albino NMRI mice (Pasteur Institute; Tehran, Iran) weighing 20-30 g were used. The animals were housed 10 per a Plexiglas cage and maintained in a room with controlled light/dark cycle (12/12 h with light beginning at 7:00 a.m.) and temperature (22 ± 2 °C), with free access to food and water. They were allowed to adapt to the laboratory conditions for at least one week before surgery. The experiments were carried out during the light phase of the photoperiod. Ten animals were used in each group of experiments and each animal was used once only. In this research, 25 groups were examined. The research adhered to the Principles of Laboratory Animal Care (NIH publication #85−23, revised in 1985)

*Surgery and cannulae implantation*

Mice were anesthetized with intraperitoneal injection of ketamine/xylazine mixture (100 and 10 mg/kg, respectively), and placed in a stereotaxic frame (Stoelting Co. USA). The skin was incised and the skull was cleaned. Two 23-gauge guide cannulae were placed (bilaterally) 1 mm above the intended site of injection according to the atlas of Paxinos and Franklin (2001). Stereotaxic coordinates for the CA1 regions of the dorsal hippocampus were AP: -2 mm from bregma, L: ±1.6 from the midline, and V: -1.5 mm from the skull surface. The guide cannulae were anchored to the skull by a jeweler’s screw and dental cement, and then two stainless steel stylets (30-gauge) were inserted into the guide cannulae to keep them patent prior to injections. All animals were allowed one week to recover from the surgery.

*Intra-hippocampal microinjections*

For drug infusions, the animals were gently restrained by a hand; the stylets were removed from the guide cannulae and a 30-gauge injection needle was inserted (1 mm beyond the tip of the guide cannula). The injection solutions were administered in a total volume of 1 μL/mouse (0.5 μL/each side) over a 60 sec period followed by an additional 60 sec to facilitate the diffusion of the drugs from the tip of the guide cannula.

*Apparatus*

The inhibitory avoidance apparatus consisted of a wooden box (30×30×40 cm height). The floor of which was consisted of stainless steel rods (29 parallel rods, 0.3 cm in diameter, set 1 cm apart). A wooden platform (4×4×4 cm) was set in the center of the grid floor. Intermittent electric shocks (1 Hz, 0.5 sec, 40 V DC) were delivered to the grid floor by an isolated stimulator (Grass S44, USA).

*Inhibitory avoidance task*

A single-trial step-down inhibitory avoidance task was used. Each mouse was gently placed on the wooden platform. When the mouse stepped down from the platform and placed all its four paws on the grid floor, the electric shock was delivered continuously for 15 sec. This training procedure was carried out between 9:00 and 13:00 h. Each mouse was placed on the platform again at 24 h after training and the step-down latency was recorded with a stopwatch as inhibitory avoidance memory. An upper cut-off time of 300 sec was set. The retention test was also carried out between 9:00 and 13:00 h.

*Drug treatment*

Ten animals were used in each experimental group. In the experiments where animals received two injections, the control groups also received two saline or vehicle injections. The drug doses for lithium and GABA_A _agents were selected according to pilot experiments and our previous studies (32-37).

*Experiment 1*

In this experiment effects of post-training administration of lithium on inhibitory avoidance memory, and then effects of pre-test administration of lithium on memory impairment induced by lithium (10 mg/kg) given after training were examined. Four groups of Animals were used in this experiment. One group of animals received post-training and pre-test saline administration. The other groups received immediate post-training lithium (10 mg/kg), and on the test day they received saline or different doses of lithium (5 or 10 mg/kg, IP) 45 min prior to the test.

*Experiment 2*

This experiment examined effect of intra-CA1 administration of GABA_A_ receptor antagonist, bicuculline, on memory retrieval by the lower dose of lithium. Nine groups of animals were used in this experiment. One group received injections of saline (10 mL/kg) both post-training and pre-test. The other eight groups of animals received lithium (10 mg/kg) after training and divided in two sets with four groups. On the test day, the animals of the first set received saline (10 mL/kg) 45 min before testing plus intra-CA1 injections of vehicle or bicuculline (0.062, 0.125 or 0.25 μg/mouse) five min before testing. The animals of the second set received LiCl (5 mg/kg) 45 min before testing plus intra-CA1 injections of vehicle or bicuculline (0.062, 0.125 or 0.25 μg/mouse) five min prior to the test.

*Experiment 3*

This experiment examined effect of intra-CA1 administration of GABA_A_ receptor agonist, muscimol, on memory retrieval by the lithium. Twelve groups of animals were used in this experiment. One group received injections of saline (10 mL/kg) both post-training and pre-test. Three groups of the animals received post-training saline, and on the test day they received muscimol (0.01, 0.03 or 0.06 μg/mouse, intra-CA1) five min prior to testing. The other eight groups of animals were received post-training lithium (10 mg/kg) and divided in two sets with four groups. On the test day, the animals of the first set received saline (10 mL/kg) 45 min before testing plus intra-CA1 injections of saline or muscimol (0.01, 0.03 or 0.06 μg/mouse) five min prior to testing. The animals of the second set received LiCl (10 mg/kg) 45 min before testing plus intra-CA1 injections of saline or muscimol (0.01, 0.03 or 0.06 μg/mouse) five min before to the test.

*Data analysis*

Because of the individual variations, the data were analyzed by one-way analysis of variance (ANOVA) for non-parametrical data (Kruskal-Wallis) followed by a two-tailed Mann-Whitney’s U-test. Then, Holmes Sequential Bonferroni correction was done for paired comparisons. The step-down latencies (as an index of inhibitory avoidance memory) for each experimental group were expressed as the median ± quartiles. In all statistical evaluations, p < 0.05 was used as the criterion for statistical significance.

## Results

*State-dependent learning induced by lithium*

**Figure 1:**
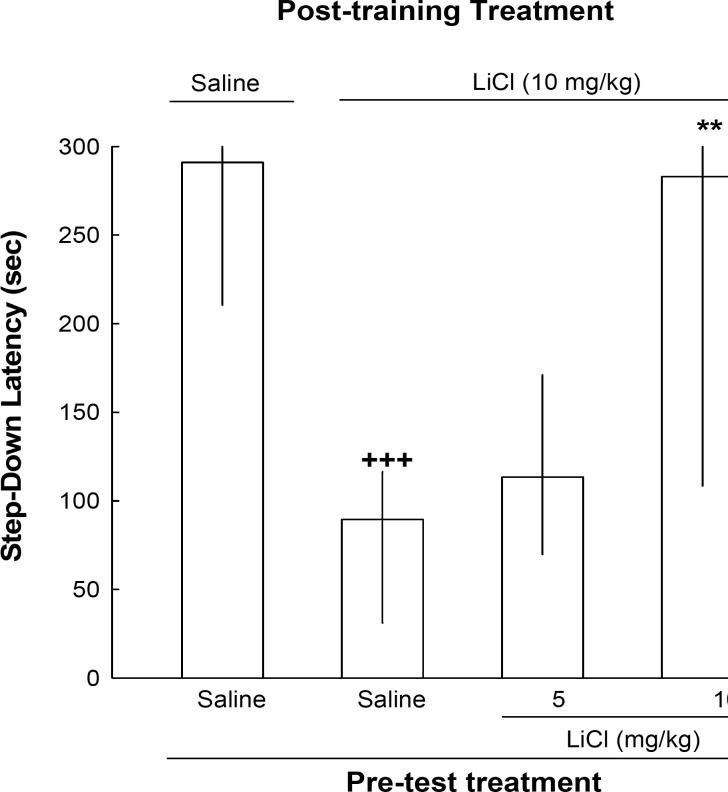
The effects of post-training and pre-test administration of lithium on step-down latency. Four groups of animals were used in this experiment. One group of animals received post-training and pre-rest injections of saline. The other groups received post-training injection of lithium (10 mg/kg). On the test day, one group of these animals received saline and the other two groups received lithium (5 or 10 mg/kg, IP), 45 min before testing. Each bar represents the median±quartiles for 10 animals. +++p < 0.001 compared to saline-saline group. ** p < 0.01 compared to lithium-saline group

As shown in Figure 1, post-training administration of lithium (10 mg/kg) decreased the step-down latency on the test day compared with saline-treated animals, and the decrease in the step-down latency due to post-training lithium was fully or partly reversed by pre-test administration of the drug [Kruskal–Wallis non-parametric ANOVA, H(3) = 17.97, p < 0.001]; suggesting state-dependent learning induced by lithium (Figure 1)

*Effect of pre-test intra-CA1 injections of bicuculline on reversal of state-dependent learning by a lower dose of lithium*

**Figure 2:**
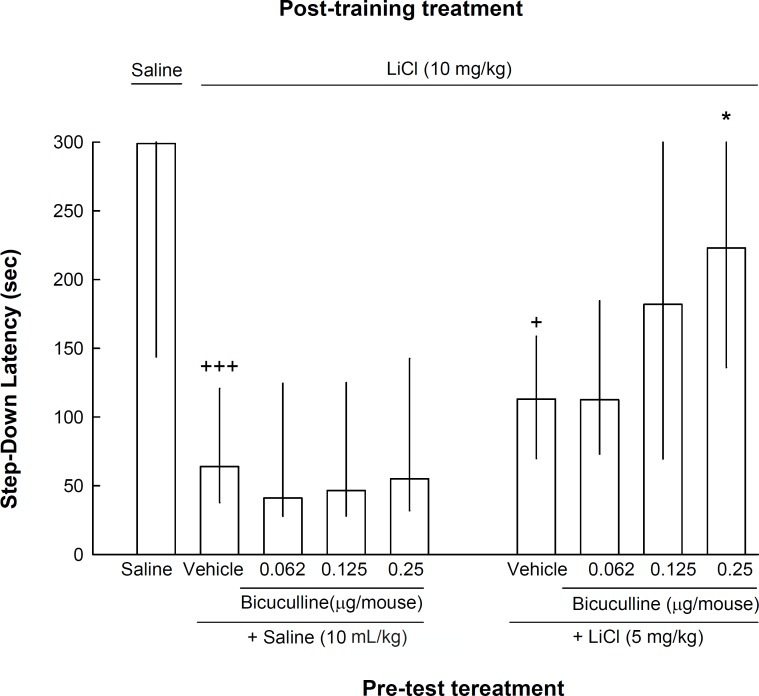
The effects of intra-CA1 administration of bicuculline, on memory retrieval by the lower dose of lithium. Nine groups of animals were used in this experiment. One group received injections of saline (10 mL/kg) both post-training and pre-test. The other eight groups of animals received lithium (10 mg/kg) after training and divided in two sets each with four groups. On the test day, the animals of the first set received saline (10 mL/kg) 45 min before testing plus intra-CA1 injections of vehicle or bicuculline (0.062, 0.125 or 0.25 μg/mouse) five min before testing. The animals of the second set received LiCl (5 mg/kg) 45 min before testing plus intra-CA1 injections of vehicle or bicuculline (0.062, 0.125 or 0.25 μg/mouse) five min prior to the test. +p < 0.05 and +++p < 0.001 compared to saline-saline group. *p < 0.05 compared to lithium-vehicle+lithium) group

The result of experiment 2 showed that pre-test administration of a lower dose of lithium (5 mg/kg) in combination with different doses of intra-CA1 administration of bicuculline (0.062, 0.125 and 0.25 μg/mouse) increased the step-down latency compared to the control group [Kruskal–Wallis non-parametric ANOVA, H(8) = 29.92, p < 0.001]. Post-hoc analysis revealed that bicuculline at dose of 0.25 μg/mouse increased the effect of pre-test lithium (5 mg/kg) on reversal of step-down latency (Figure 2). 

*Effect of muscimol on state-dependent learning induced by lithium*

**Figure 3:**
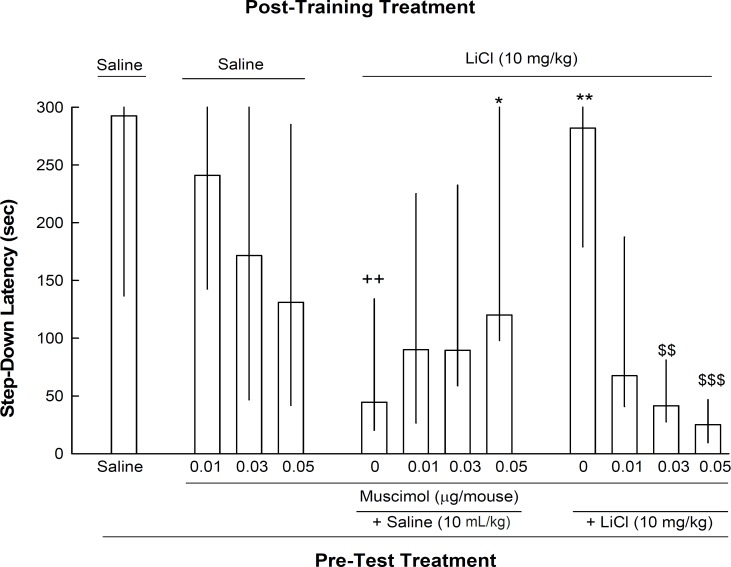
The effects of intra-CA1 administration of muscimol, on memory retrieval by lithium. Twelve groups of animals were used in this experiment. One group received injections of saline (10 mL/kg) both post-training and pre-test. Three groups of the animals received post-training saline and on the test day they received muscimol (0.01, 0.03 or 0.06 μg/mouse, intra-CA1) five min prior to testing. The other eight groups of animals received post-training lithium (10 mg/kg) On the test day, these eight groups divided in two sets of four groups and each four groups received intra-CA1 injections of saline or muscimol (0.01, 0.03 or 0.06 μg/mouse) plus either saline (10 mL/kg) or LiCl (10 mg/kg). ++p < 0.01 compared to saline-saline group. * p < 0.05 and ** p < 0.01 compared to lithium-(saline-saline) group. $$ p < 0.01 and $$$ p < 0.001 compared to lithium-(saline-lithium10 mg/kg) group

The result of experiment 3 revealed that pre-test administration of muscimol had no significant effect on step-down latency of post-training saline treated animals. On the other hand, although muscimol (0.06 μg/mouse) reversed the decrease of step-down latency induced by post-training lithium (10 mg/kg), but it disrupted reversal effect of pre-test lithium (10 mg/kg) on state-dependent learning [Kruskal–Wallis non-parametric ANOVA, H(11) = 41.47, p < 0.001] (Figure 3).

## Discussion

Consistent with our previous studies (31, 34), the present data showed that post-training administration of lithium decreased step-down latency of inhibitory avoidance task in mice, which was fully or partly reversed by pre-test administration of the drug. This effect of lithium on the step-down latency seems to be due to state-dependent learning (31-34). In state-dependent learning when pre- or post-training administration of a drug decreases memory for a task, administration of the drug prior to testing reinstates the memory for the task (38-40). State-dependent learning has been reported for many drugs and hormones (41-44). With the idea of state-dependent learning, in animals which received post-training lithium and pre-test saline in the present study, the decrease of the step-down latency may be due to this fact that the animals did not receive lithium before the test. However, the exact mechanism of state-dependent learning induced by drugs, including lithium, will require more investigations.

It has been reported that lithium which is used in treatment of bipolar disorders may increase GABA signaling (35). On the other hand, several lines of experimental data indicate that GABA_A_ receptors are involved in learning and memory processes (3, 12, 45, 46). Therefore, in the present research we examined effects of intra-CA1 injections of the GABA_A_ receptor agents on state-dependent learning induced by lithium. At first, we injected different doses of lithium with the GABA_A_ receptor antagonist and agonist (bicuculline and muscimol, respectively) and we observed that bicuculline and muscimol showed different effects by themselves and in combination with lithium.

The results of the present study showed that intra-CA1 injections of the GABA_A_ receptor antagonist, bicuculline before testing partly increased the effect of lower dose of lithium on reinstatement of the step-down latency. A positive effect for bicuculline on memory has been also reported in other studies. It has been reported that post-training administration of bicuculline increased memory retention in rat (46). Bicuculline, when administered into the amygdala after training, blocked benzodiazepine-induced amnesia (47). However, lower doses of bicuculline induced amnesia at both low and high footshock intensities (45).

The present results also revealed that in the post-training saline treated animals, intra-CA1 injections of the GABA_A_ receptor agonist, muscimol before testing had no significant effect on the step-down latency. In the post-training lithium treated animals, pre-test intra-CA1 injection of muscimol reversed the decrease of step-down latency due to post-training lithium. However, pre-test intra-CA1 injection of the same dose of muscimol disrupted state-dependent learning induced by lithium. 

It has been also shown that post-training intrahippocampal injection of muscimol decreased memory retention of inhibitory avoidance in rats dose-dependently (46). The impairment and enhancement of retention of an inhibitory avoidance task by muscimol and bicuculline, respectively, was blocked in animals with amygdala and dorsal hippocampus lesions (48). It has been reported that amygdaloid GABA neurotransmission or mRNA transcription controls footshock-associated fear using a fear passive avoidance task (49). Unilateral microinjection of midazolam into the basolateral amygdala (via a GABAergic mechanism) reduced avoidance responses (50). The above cited data may further support that the GABA_A_ receptors in the hippocampus are important for inhibitory avoidance memory. It has been reported that immediate post-training systemic injections of bicuculline improved and muscimol impaired retention of inhibitory avoidance task in CD1 mice, which did not reversed by pre-test administration of the same drugs (51). According to the present results and the above cited data it seems that the effects of post-training administration of bicuculline and muscimol on retention are not state dependence.

Taken together our results about effects of bicuculline and muscimol on inhibitory avoidance memory are consistent with other reports that bicuculline showed a positive effect but muscimol induced a negative effect. Since pre-test administration of bicuculline into the CA1 region of the hippocampus could increase the effect of pre-test lithium on reversal of memory, one may propose that sate-dependent learning induced by lithium to be dependent at least partly on a GABAergic mechanism in the hippocampus. In addition, state-dependent learning induced by lithium disrupted due to pre-test injections of muscimol which may further support the involvement of GABA_A_ receptors of the hippocampus in effects of lithium on inhibitory avoidance memory.
